# Noninvasive Sweat-Lactate Biosensor Emplsoying a Hydrogel-Based Touch Pad

**DOI:** 10.1038/s41598-019-46611-z

**Published:** 2019-07-12

**Authors:** Kuniaki Nagamine, Taisei Mano, Ayako Nomura, Yusuke Ichimura, Ryota Izawa, Hiroyuki Furusawa, Hiroyuki Matsui, Daisuke Kumaki, Shizuo Tokito

**Affiliations:** Research Center for Organic Electronics (ROEL), Yamagata University, 4-3-16 Jonan, Yonezawa, Yamagata 992-8510 Japan

**Keywords:** Electrochemistry, Electrical and electronic engineering

## Abstract

This study is the first report demonstrating proof-of-concept for a hydrogel-based touch sensor pad used for the non-invasive extraction and detection of sweat components. The sensor device was composed of an electrochemical L-lactate biosensor covered with an agarose gel in a phosphate buffer saline. When human skin contacts the agarose gel, L-lactate in sweat was continuously extracted into the gel, followed by *in-situ* potentiometric detection without controlled conditions. This novel type of sweat sensor is expected to enable the simple, non-invasive daily periodic monitoring of sweat biomarkers for advanced personal healthcare methods in the future.

## Introduction

Non-invasive diagnostic sensors for biomarkers are generally required as an alternative to blood-based diagnoses for the next generation of advanced healthcare. Notably, the externally-excreted bodily fluids such as tears, saliva, urine, and sweat have been considered as potential targets because recent metabolomic and proteomic analysis has revealed the existence of some biomarkers partitioned from blood or interstitial fluids^1–6^. Among these bodily fluids, sweat secreted through the skin from eccrine, apocrine, apoeccrine, or sebaceous glands are examples of easily accessible source of biomarkers^7^, however, the complexity of sweat composition including partial blood composition partitioned from interstitial fluids, various secretions from sweat gland cells, and the metabolites from resident inhabitant bacteria on human skin makes it difficult to identify the origin of biomarkers^8^. Moreover, it is challenging to perform subsequent analyses in small amount of available sweat to a periodic or continuous manner. Wearable-type of tattoo- and microfluidics-based biosensors have enabled *in-situ* and real-time sampling and monitoring of sweat biomarkers^9–15^. However, it has been necessary to induce continuous perspiration under special conditions such as with exercise, in a high-temperature environment^16^, or with the use of cholinergic agonist-assisted perspiration^15,17,18^. These circumstances are not practical in the application of sweat sensors for daily health diagnostics.

Tsuda *et al*. proposed a straightforward and periodic sweat sampling method^19,20^. Human skin was exposed to 1 vol% aqueous ethanol to extract the sweat component into aqueous ethanol. They emphasized that the addition of ethanol into water induces a smaller surface tension of the solution to the skin allowing the solution to penetrate the sweat gland effectively. This sweat collection method has also been used for recent proteomic and metabolomic analyses^21–24^. K. Hooton and L. Li suggested clear correlation between real and extracted sweat^23^, and M. Tsunoda *et al*. suggested clear correlation between plasma and extracted sweat component concentration^25^. However, the extracted sweat components were quantified using separately prepared analytical techniques, such as liquid chromatography and mass spectrometry^19–25^. For daily diagnostics, a sweat extraction system should be combined with biosensors to detect the extracted sweat components locally and *in-situ*.

**Figure 1:**
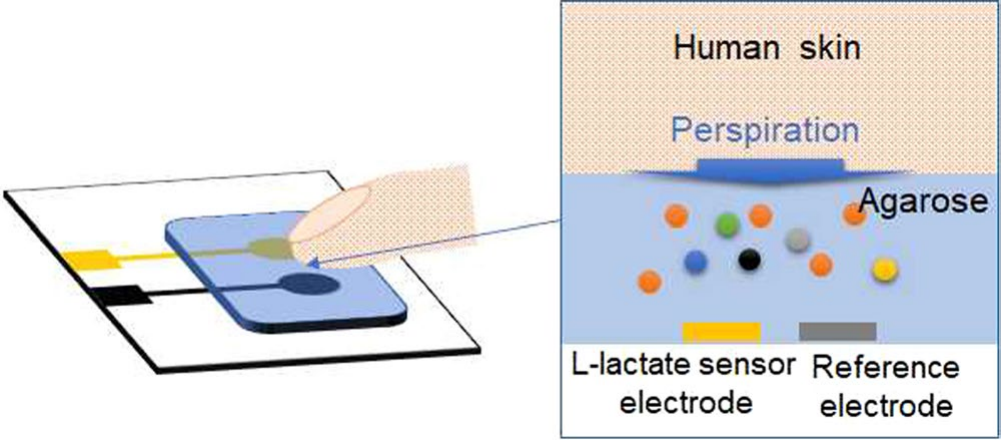
Schematic view of the hydrogel-based chemical touch sensor for sweat analysis.

This study reports on a hydrogel-based touch sensor pad for the non-invasive extraction and detection of sweat components (Fig. 1). The sensor was composed of electrochemical biosensing working and reference electrodes fully covered with an agarose hydrogel including a sweat-extraction solution, Dulbecco’s Phosphate Buffer Saline (DPBS). The sweat components can be easily and continuously extracted from human skin contacted with the agarose hydrogel, followed by *in-situ* detection. In this study, an enzyme-based L-lactate sensor was fabricated as a model because L-lactate is included in sweat in relatively high concentrations. The difficulty in realizing this type of sweat sensor is to stabilize the electrical potential of the reference electrode in the sweat-extraction solution, whereby composition is continuously changing during sweat extraction. We found that the DPBS extraction solution enables continuous extraction and stabilizes the electrochemical potential of Ag/AgCl reference electrode during sweat extraction and detection. This new type of touch sensor pad integrated with biosensors will enable non-invasive and daily periodic monitoring of sweat biomarkers without harsh exercise-, environmental temperature control, and cholinergic agonist-assisted perspiration.

## Results and Discussion

**Figure 2:**
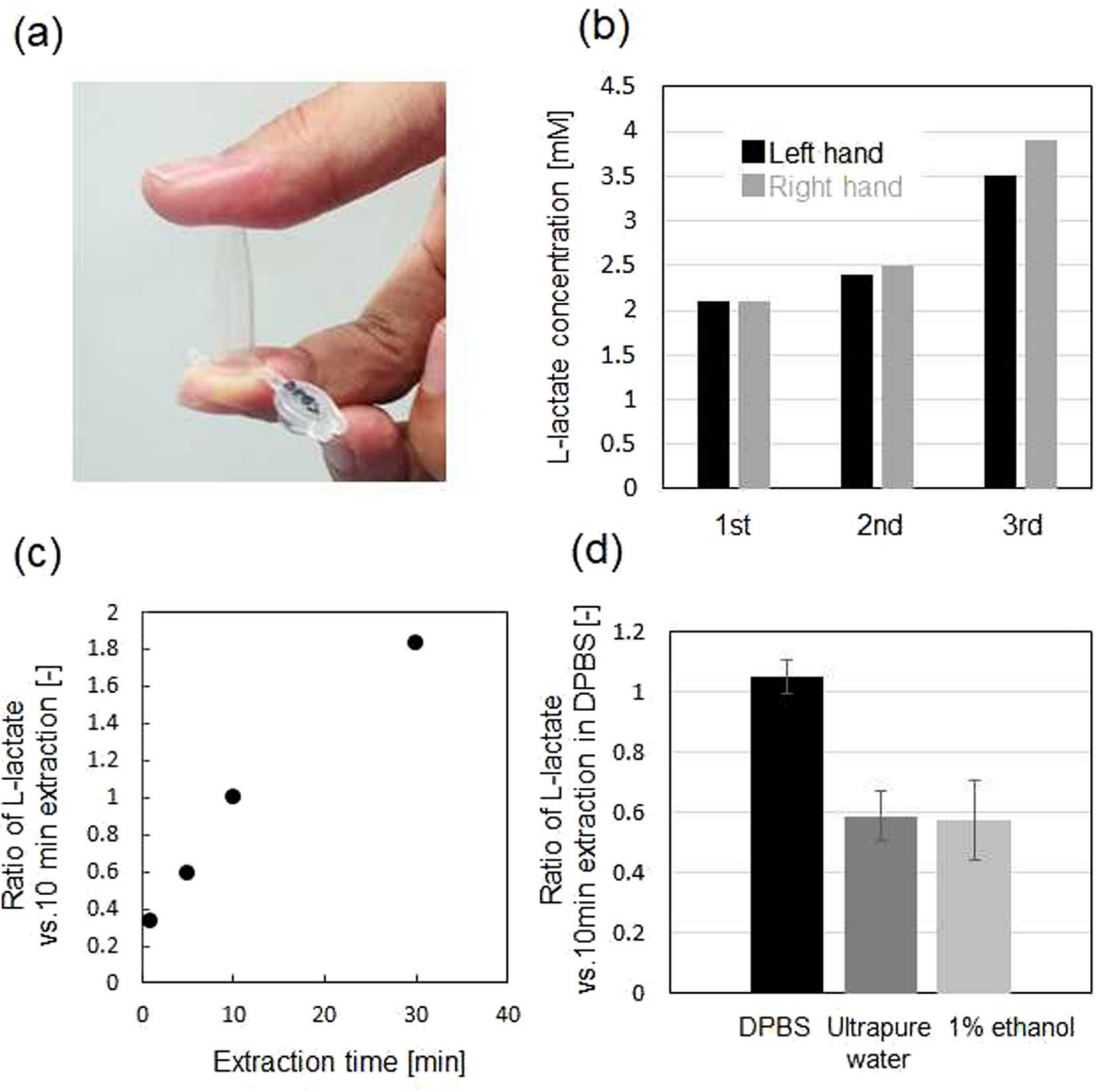
(**a**) Photograph of sweat sampling method from the side of a subject’s forefinger. (**b**) L-lactate concentration in the DPBS collected for fingers from the right (gray square) and left hand (black square), respectively. (**c**) Changes in extracted L-lactate concentration depending on the sweat extraction time. (**d**) Extracted L-lactate concentration using three kinds of extraction solution, DPBS, Milli-Q water, and 1 vol% ethanol-water.

We first carried out a study to replicate the sweat extraction methods proposed by Tsuda *et al*.^19,20,25^ in order to optimize the composition of the sweat extraction solution. Figure 2 shows the results of quantification of L-lactate in the sweat extracted from the side of the subject’s forefinger. After washing the subject’s right and left-hand forefingers with running 1 vol% ethanol-water for 15 s, a 0.6 mL-volume of microcentrifuge tubes containing 30 μl of DPBS was put on the washed fingers simultaneously as DPBS was contacted with skin surface for 10 min at room temperature as shown in Fig. 2a. The L-lactate concentration in the DPBS collected from the skin surface was quantified using a commercially-available L-lactate sensor. Figure 2b shows L-lactate concentration in the DPBS gathered from the subject’s forefingers for right (gray square) and left hand (black square), respectively. The sampling process was conducted three times for the same subject at different times in the same day. The concentrations of L-lactate extracted from the subject’s right and left-hand fingers in each sample had very similar values. Tsuda *et al*. also suggested that the amount of sweat secreted from fingers was almost the same when a subject was under stimulation by deep breathing^19^. L-lactate concentration was different for sweat extracted at three different times which was probably due to circadian changes in sweat L-lactate concentration or perspiration rate during the day. Figure 2c shows the variation in extracted L-lactate concentration depending on the time of sweat extraction. To avoid the influence of circadian changes on sweat L-lactate concentration, the proportion of L-lactate concentration obtained from the right- and left-hand forefingers was calculated. Specifically, the right-handed finger was exposed continuously to DPBS for 10 min, while that from the left-hand was exposed to DPBS for 0, 5, 10, 30 min at the same time. When the right and left-hand fingers were exposed to DPBS for 10 min, the ratio of L-lactate concentration was about one as shown in Fig. 2b. However, when the extraction time for the left-hand finger was increased, the proportion of extracted L-lactate increased, suggesting continuous perspiration in the DPBS extraction solution. These results are supported by a previous study demonstrating constant perspiration in a solution within which the osmotic pressure was controlled by adding salts and saccharides^26^. Figure 2d examines the effect of the kinds of extraction solution, DPBS, ultrapure water, and 1 vol% ethanol-water for a representative subject. Further, the forefinger of right hand was continuously exposed to DPBS for 10 min, while that of left hand was exposed to different extraction solution for 10 min at the same time, followed by calculating the ratio of extracted L-lactate concentration from both hands. The amount of extracted L-lactate in DPBS was higher than that in ultrapure water and 1 vol% ethanol-water. The L-lactate in DPBS extracted from another subject was also higher than that of ultrapure water, while similar to that extracted in 1 vol% ethanol-water (Supplementary Fig. S1). 1 vol% ethanol-water was proposed by Tsuda *et al*. as an effective extraction solution because the addition of ethanol into water induces decrease of surface tension of the extraction solution to the skin^19^. However, the ethanol-water was not suitable for our device because the ethanol-water extraction solution will affect the enzymatic activity of the L-lactate biosensor. Besides, evaporation of ethanol from an extraction solution and the detection process is a separate concern. However, the L-lactate extracted into Milli-Q water was about half of that in DPBS and 1 vol% ethanol-water. The previous study suggested the suppression of perspiration in ultrapure water due to the closing of sweat glands from mechanical swelling of the skin^27^. These factors taken together, DPBS was selected as an extraction solution in the following study.

**Figure 3:**
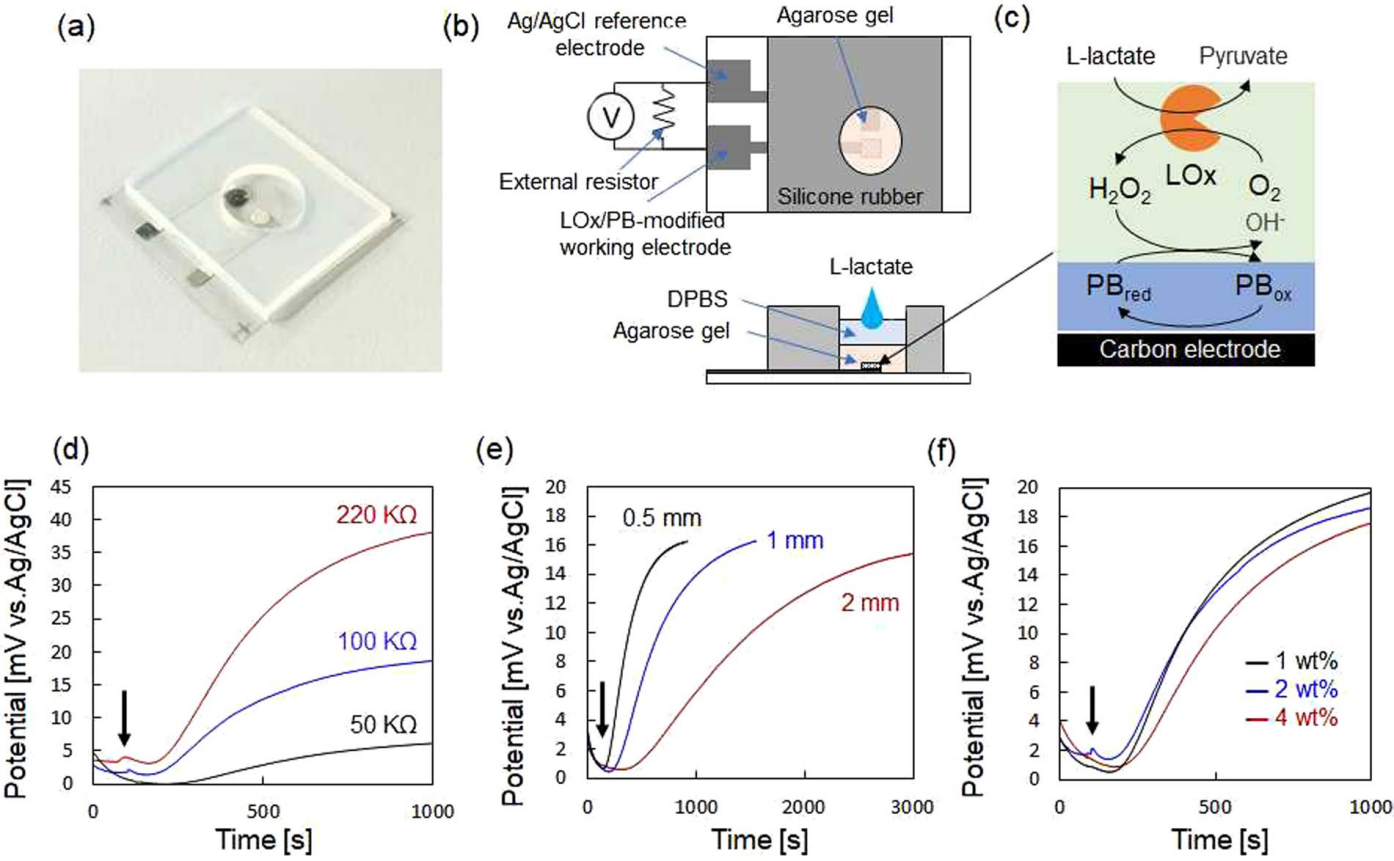
(**a**) Photograph of the hydrogel-based L-lactate sensor. (**b**) Construction of the L-lactate sensor. (**c**) Image of the enzymatic reaction on the working electrode. Potentiometric response of the sensor upon injection of 0.5 mM L-lactate for different configurations: (**d**) External resistors of 50 kΩ (black), 100 kΩ (blue), and 220 kΩ (red). (**e**) Agarose gel pad thicknesses of 0.5 mm (black), 1 mm (blue), and 2 mm (red). (**f**) Agarose gel concentrations of 1 wt% (black), 2 wt% (blue), and 4 wt% (red). The black arrows in (**d**–**f**) represent the time of L-lactate injection.

**Figure 4:**
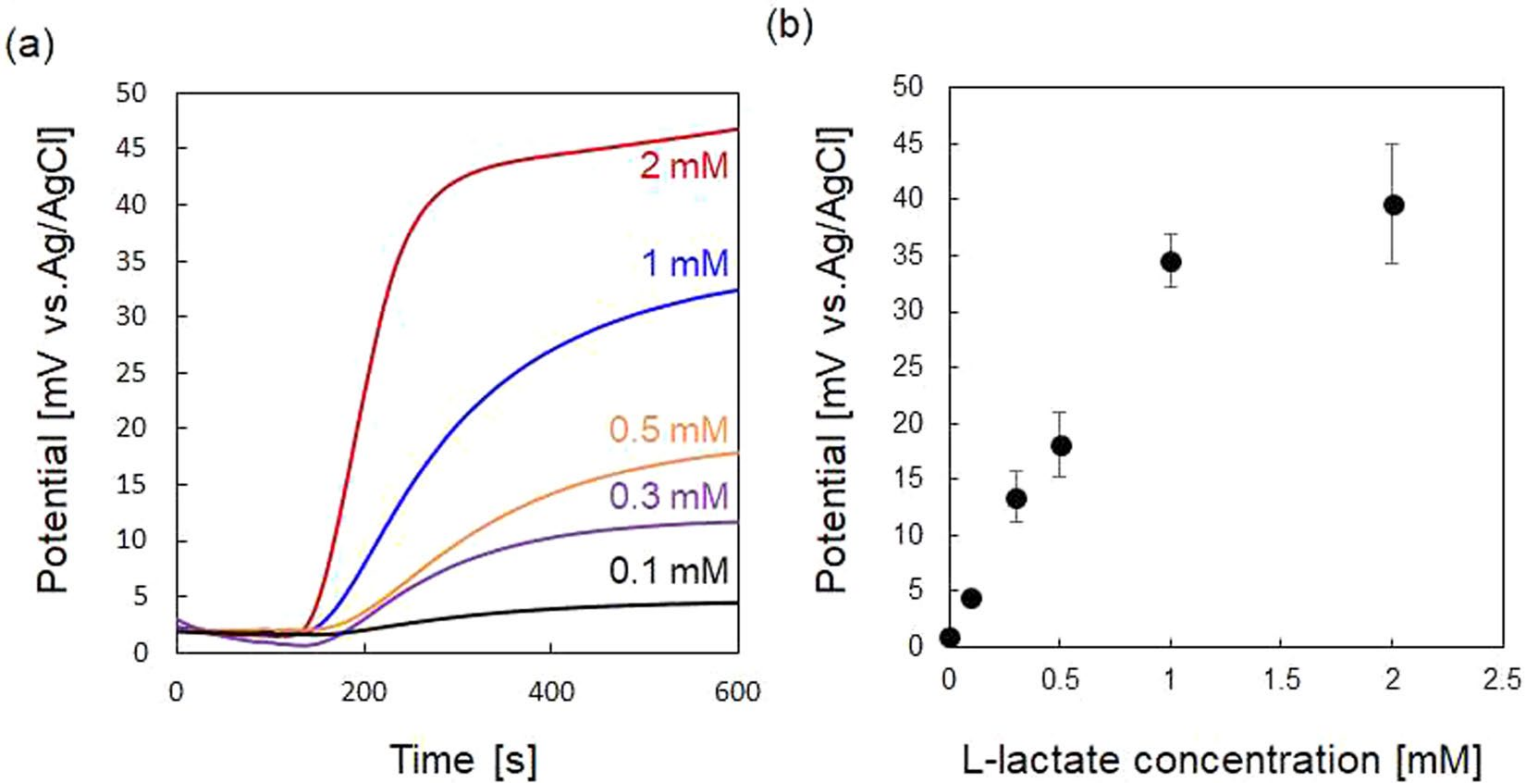
(**a**) Potentiometric response of the hydrogel-based L-lactate sensor upon injection at 100 s for different L-lactate concentrations. (**b**) Change in potential difference between the LOx/PB-modified electrode and bare Ag/AgCl reference electrode at various L-lactate concentrations in DPBS. The external resistor was 100 kΩ. The agarose hydrogel concentration and thickness of pad were 4 wt% and 0.5 mm, respectively.

Figure 3 shows the construction of the hydrogel-based sweat sensor and its characteristics. The lactate oxidase (LOx)/Prussian blue (PB)-modified working electrode, and bare Ag/AgCl reference electrode on the polyethylene naphthalate (PEN) film substrate were covered with a cylindrical form of agarose gel in a DPBS extraction solution (6 mm in diameter) surrounded by a silicone rubber sheet. The agarose gel with 0.5 mm, 1 mm, or 2 mm thickness was covered with same volume of DPBS solution, and the concentrated L-lactate in DPBS was drop-deposited into the DPBS solution to be uniformly diffused into the agarose gel as shown in Fig. 3b. L-lactate was oxidized by LOx to generate hydrogen peroxide, which oxidize reduced from PB (PB_red_) to an oxidized form (PB_ox_) as shown in Fig. 3c. The terminal lines of LOx/PB-modified working electrode and bare Ag/AgCl reference electrode were short-circuited with an external resistor to allow reversible potentiometric monitoring^28^. The potential difference across the external resistor was measured using an electrochemical analyzer operated in the open-circuit potential mode. Figure 3d shows the potentiometric response of the sensor against 0.5 mM L-lactate for different values of external resistance (50 kΩ, 100 kΩ, and 220 kΩ). The concentration of agarose gel was 2 wt%, and the agarose gel thickness was 1 mm. After waiting for several minutes to obtain a steady-state potentiometric response, the concentrated L-lactate was applied on the agarose gel at Time = 100 s to be final L-lactate concentration of 0.5 mM. The time to obtain steady-state potentiometric response before injection of L-lactate was shortened with a decrease of external resistivity. The potential difference started to increase about 100 s after L-lactate injection, followed by approaching to steady-state. 90% response time was almost the same among different resistors: 650 s for 50 kΩ, 550 s for 100 kΩ, and 650 s for 220 kΩ, respectively. The steady-state potential difference after injection of L-lactate decreased with the decrease of external resistance because of short-circuiting between the LOx/PB-modified electrode and the Ag/AgCl reference electrode^28^. In the following study, the value of external resistivity was set to 100 kΩ to obtain quick stabilization of potential difference before L-lactate injection with sustaining significant potentiometric response. Figure 3e shows the potentiometric response of the L-lactate sensor with different thicknesses of 2 wt% agarose gel in DPBS against 0.5 mM of L-lactate. The time delay between the injection of L-lactate and the onset of a potentiometric response increased slightly with increase of agarose gel thickness: 48 s for 0.5 mm thick, 100 s for 1 mm thick, and 224 s for 2 mm thick, respectively, while the value of steady-state potential difference was almost same regardless of agarose gel thickness. The rate of potentiometric response also decreased with an increase agarose gel thickness: 90% response time was 400 s for 0.5 mm thick, 850 s for 1 mm thick, and 1900 s for 2 mm thick, respectively. These results suggested that one of the rate-limiting steps determining the potentiometric response rate was diffusion rate of L-lactate in the agarose gel. Figure 3f shows the potentiometric response of the L-lactate sensor with different concentration of agarose gel (1, 2, and 4 wt%) in DPBS. The delay time and the 90% response time were nearly the same regardless of the agarose gel concentration. 90% response time was achieved for 600 s for 1 wt%, 500 s for 2 wt%, and 600 s for 4 wt%, respectively. These results suggest that the concentration of agarose gel did not seriously affect the diffusion rate of L-lactate in the gel most likely because the agarose gel has a relatively larger pore structure with a diameters ranging from 0.1 to 1 µm than the molecular size of L-lactate (7.5 Å)^29^. These results also support our consideration that the rate-limiting step of the sensor response was diffusion rate of L-lactate in the agarose gel. In this study, the concentration and thickness of the agarose gel were set to 4 wt% and 0.5 mm thickness to obtain mechanical stability and faster potentiometric response rate. Figure 4a,b show the potentiometric response of the L-lactate sensor against various L-lactate concentration and dose-response curve against L-lactate concentration, respectively. The sensor exhibited a typical enzymatic response against L-lactate concentration with good reproducibility (n = 3), suggesting that there is little difference in electrochemical characteristics between the fabricated sensor devices.

During sweat extraction, various types of ions other than L-lactate are extracted into the extraction solution, that would induce a change in the electrochemical measurement conditions. For example, pH change will affect enzymatic activity, probably resulting in low-reproducibility of the L-lactate sensor response. The difference in pH of the DPBS extraction solution before and after 10 min of sweat extraction was measured as 0.2, whereas 1.0 of pH change was measured for ultrapure water-based extraction. These results suggest that DPBS is effective for not only continuous sweat extraction, but also stabilizing pH in the extraction solution. Another concern is the adverse effect of a change in Cl^−^ ion concentration on the electrical potential stability of bare Ag/AgCl reference electrode. Na^+^ ion concentration, which is included in human sweat in almost the same level as the Cl^−^ ions^30^, extracted in DPBS was measured using a commercially available Na^+^ ion meter. Change in Na^+^ ion concentration during 10 min of sweat extraction was less than 10 mM. Assuming the same level of Cl^−^ is extracted into DPBS during 10 min of sweat extraction, the potential change of the bare Ag/AgCl electrode against the Ag/AgCl reference electrode was only 1.2 mV (Supplementary Fig. S2). This is because DPBS initially contains a high concentration of Cl^−^ (139.7 mM), change of Cl^−^ ion concentration from 139.7 mM to 149.7 mM induces slight potential change according to Nernst equation. As described below, this small change in potential for the bare Ag/AgCl reference electrode had a negligible effect on the L-lactate sensor response.

**Figure 5:**
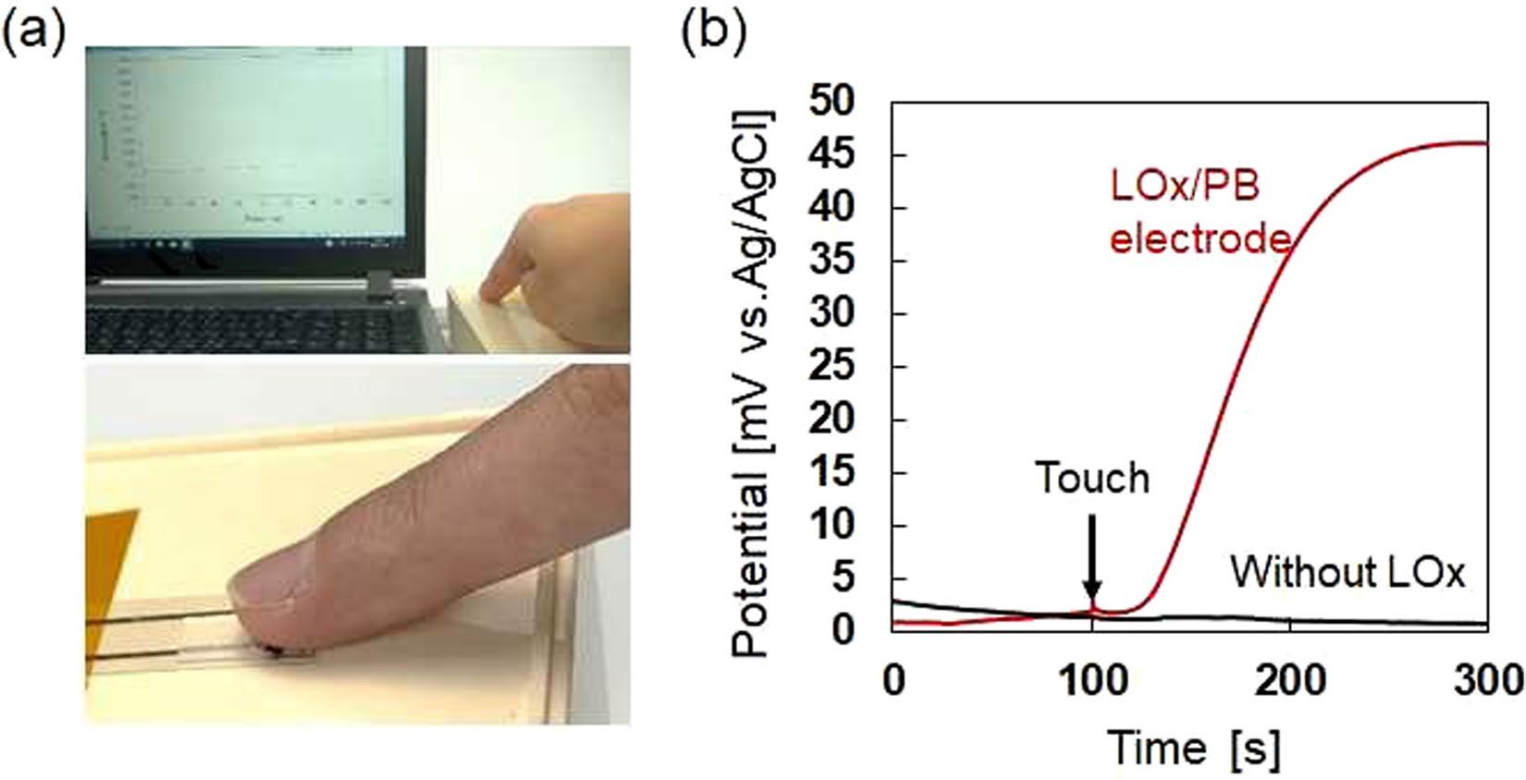
(**a**) Photographs of a human sweat extraction and detection experiment using the hydrogel-based L-lactate sensor. (**b**) Potentiometric response of the sensor electrodes with and without LOx when the ball of the subject’s forefinger contacted the agarose gel surface at 100 s. The external resistor was 100 kΩ. The agarose hydrogel pad concentration and thickness were 4 wt% and 0.5 mm, respectively.

Finally, we demonstrated the extraction and detection of a subject’s sweat L-lactate using the present device in Fig. 5. The terminal lines of the LOx/PB-modified electrode and the Ag/AgCl reference electrode were short-circuited with a 100 kΩ external resistor. The concentration of agarose gel was 4 wt%, and the gel thickness was 0.5 mm. After stabilizing the potentiometric response of the sensor, the ball of subject’s finger was placed onto the surface of agarose gel for 30 s (Fig. 5a), and then removed from the gel. During this process, the potential difference was continuously monitored using the electrochemical analyzer operated in the open-circuit potential mode. Figure 5b shows the potentiometric response of the sensor when the ball of subject’s forefinger was put on the agarose gel surface. After 20 s of time lag for diffusion of extracted L-lactate in the gel, the potential difference started to increase, followed by approaching steady-state as shown in Supplementary Movie S1. The detected potentiometric response was corresponding to about 2 mM of L-lactate calculated from Fig. 4b. Without LOx, no potentiometric response was observed upon touching the gel with the subject’s forefinger (Fig. 5b), suggesting that the extracted sweat compounds except for L-lactate did not affect the sensor response. As discussed above, various kinds of ions including Cl^−^ also diffused into the gel during sweat extraction, and could affect the potential stability of the bare Ag/AgCl reference electrode. A stable sensor electrode without LOx having no potentiometric response suggests a negligible change in the electrical potential of the Ag/AgCl reference electrode during sweat extraction. One of the advantages of using an agarose gel pad is arbitral controllability of its volume to tune concentration of extracted sweat components in the gel within the detection range of biosensors with sacrificing detection speed.

These type of sweat sensor electrodes can be combined with a self-made potentiometer circuit film and a commercially available battery film to be worn on the human body so that the agarose gel contacts the skin. Supplementary Fig. S3 shows a demonstration of real-time monitoring of sweat L-lactate extracted from a human forearm. Bluetooth Low Energy (BLE) protocols are used for wireless communication. It was noteworthy that the current response could be monitored immediately after wearing the sensor on human forearm even when the evidence of sweating was not visible to the naked eye. Previous wearable sweat sensors could not monitor the electrical signal before the release of sweat because an electrical circuit is not formed between dry working and reference electrodes. To our knowledge, this is the first report of a non-invasive and straightforward sweat component extraction and detection method using a portable and wearable biosensing system without any perspiration assistance such as exercise, environmental temperature control, or requiring a cholinergic agonist.

## Conclusions

This study demonstrates a hydrogel-based chemical touch sensor for *in-situ* sweat component analysis. This device showed potentiometric response against sweat L-lactate easily extracted from human sweat gland without harsh exercise-, environmental temperature control-, and cholinergic drug-assisted perspiration. On the other hand, the signal detected using this device did not reflect the actual L-lactate concentration in sweat, but just the concentration extracted in the agarose gel pad. The extracted L-lactate concentration can be changed depending on not only change of L-lactate concentration in sweat, but also the shift in perspiration rate. Besides, skin surface temperature also affects the electrochemical biosensor response. One of our next goals is to integrate a perspiration rate sensor and a temperature controller into the present chemical touch sensor to quantify the sweat analytes.

Another issue in this device is the origin of lactate extracted in the agarose gel pad. Some previous reports described that the extracted components are transdermally transported into the extraction solution with regardless of perspiration^31,32^, while the other reports suggested clear correlation between real and extracted sweat^23^. Therefore, we have to continue studying the correlation between real and extracted sweat components concentration with careful.

Also, we did not quantitatively evaluate the L-lactate sensor electrochemical characteristics, such as detection limit, detection range, and lifetime. These characteristics should be quantified and tuned depending on target analyte. The primary purpose of this study was to demonstrate feasibility using a model target analyte of L-lactate included in human sweat in high concentration. In a future study, we will apply the present sensor to the other sweat analytes such as D-glucose (diabetes biomarker), electrolytes (heat stroke biomarker), ammonium ion (anaerobic exercise biomarker), and some cytokines (immune response biomarker) to evaluate the sensor characteristics in more detail.

## Methods

### Sweat extraction and quantification

Sweat extraction was carried out by following a previously proposed method with minor modification^19,20,25^. The ball of subject’s forefinger was cleaned with running 1 vol% ethanol-water for 15 sec. 30 μl of Dulbecco’s phosphate buffered saline (DPBS, 2.7 mM KCl, 137.0 mM NaCl, 1.5 mM KH_2_PO_4_, 8.1 mM Na_2_HPO_4_, pH 7.4) was put into a 0.6 mL of microcentrifuge tube (ASONE, 6.0 mm in diameter). The microcentrifuge tube was put on the washed skin surface as DPBS was contacted with skin surface for an arbitrary amount of time. After that, the components in the collected DPBS was quantified using commercially available L-lactate sensor (Lactate pro2 LT-1730, Arkray) or Na^+^ ion meter (Horiba, LAQUAtwin Na-11).

### Fabrication of the sweat L-lactate biosensor

Figure 3b shows the structure of the sweat L-lactate sensor. A polyethylene naphthalate (PEN) film substrate (125 μm thickness) was used as the substrate. A 150-nm-thick of parylene (KISCO, diX-SR) layer was prepared on the PEN film by chemical vapor deposition. A silver nanoparticle ink in a hydrocarbon-based solution (Harima Chemicals, NPS-JL) was printed as working and reference electrode patterns using an inkjet printer (Fujifilm, Dimatix DMP2831). During the inkjet printing process, the temperatures of the substrate and cartridge were kept at 50 °C and 35 °C, respectively. The Ag electrode patterns had an active area of 2 mm × 2 mm for the working electrode and 2 mm × 2 mm for the reference electrode. The lead line patterns for the working and reference electrode were insulated by coating a fluoropolymer (5 wt%, DuPont, Teflon AF1600) layer in Fluorinert (3 M, FC-43), followed by annealing for 30 min at 60 °C in ambient air. The carbon-graphite ink, including a redox compound of Prussian Blue (PB-carbon) (C2070424P2, Gwent Inc.), and the Ag/AgCl paste (GWENT) were painted on the active area of working and reference electrode pattern through a stencil sheet, respectively, and dried for 15 min at 80 °C.

A 10 µL solution composed of a mixture of 10 U µL^−1^ lactate oxidase (LOx, 80 units mg^−1^, TOYOBO Co., Ltd. LOx was used without further purification.) in 100 mM phosphate buffer (pH 7.4, Nakalai Tesque) and 0.1 wt% chitosan (Junsei Chemical) in 50 mM HCl (pH 5.4) in the ratio 1.4:10 was drop-deposited on the PB-carbon area and dried for 1 h at 30 °C. The resulting working electrode was washed in stirred DPBS for 1 h to remove the LOx loosely bound to the chitosan gel. A silicone rubber sheet (ASONE) with a 6 mm diameter through-hole was attached to the electrode film as to expose the active area of working and reference electrodes to the through-hole. A low melting point agarose (Nakalai Tesque) prepolymer solution in DPBS was drop-deposited onto the through-hole in the silicone sheet while sustaining the temperature of the prepolymer solution to 30 °C, and incubated for 10 min at room temperature to induce gelation.

Figure 3b shows the setup for potentiometric measurement using the agarose hydrogel-covered L-lactate sensor electrodes. The terminal lines of LOx-immobilized PB-carbon electrode (LOx/PB-modified electrode) and bare Ag/AgCl reference electrode were short-circuited with an external resistor^28^. The potential difference across the external resistor was monitored using an electrochemical analyzer (ALS model 602E. BAS Inc.) operated in the open-circuit potential mode (input impedance: 1 × 10^12^ Ω). For evaluation of the sensor response against an L-lactate solution, the surface of agarose gel was covered with DPBS measurement solution with same volume as the agarose gel to induce uniform diffusion of L-lactate into the gel. Concentrated L-lactate dissolved in DPBS was injected in the measurement solution to induce enzymatic reaction at the LOx/PB-modified electrode. The temperature of the measurement solution was kept at 30 °C using a hot plate.

### Human sweat sampling and measurement using the biosensor

The terminal lines of LOx/PB-modified electrode and Ag/AgCl reference electrode described above were short-circuited with a 100 kΩ external resistor. The potential difference across the external resistor was measured using an electrochemical analyzer operated in the open-circuit potential mode. The subject’s skin surface was cleaned with running 1 vol% ethanol-water for 15 s just before experiment. After stabilizing the potentiometric response of the sensor, the side of subject’s forefinger was placed on the surface of agarose gel for 30 s, and them removed from the gel. During this process, the potential difference was continuously monitored at room temperature.

### Ethical approval

All procedures performed in studies involving human participants were in accordance with the standards of Ethics Committee of Faculty of Medicine, Yamagata University. All the experimental protocols involving human participants were approved by Ethics Committee of Faculty of Medicine, Yamagata University (Approval No. 30-7). Before carrying out the experiments, the purpose of this study was explained to subjects who signed the university-approved informed consent form.

## Supplementary information

Movie S1

Supplementary Information

## Data Availability

All data generated or analysed during this study are included in this published article and its Supplementary Information Files.
