# Supplementary material for: Noninvasive Sweat-Lactate Biosensor Emplsoying a Hydrogel-Based Touch Pad
Source: Sci Rep. 2019 Jul 12;9:10102. doi: 10.1038/s41598-019-46611-z (PMC6626002; doi:10.1038/s41598-019-46611-z)
Supplement: Supplementary file 2 — Supplementary Information [file 41598_2019_46611_MOESM2_ESM.pdf]

Supplementary Information

Noninvasive Sweat-Lactate Biosensor Employing a Hydrogel-Based Touch Pad

Kuniaki Nagamine*, Taisei Mano, Ayako Nomura, Yusuke Ichimura, Ryota Izawa, Hiroyuki Furusawa, Hiroyuki Matsui, Daisuke Kumaki, Shizuo Tokito*

Supplementary Information

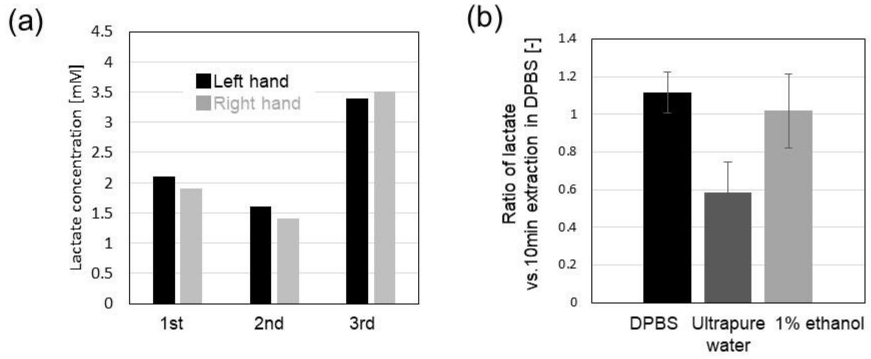

Figure S1.

Sweat extraction experiments applied on a different subject than the one used in of Fig. 2. (a) L-lactate concentration in the DPBS collected from subject's fingers for the right (gray square) and left hand (black square), respectively. (b) Extracted L-lactate concentrations using three kinds of extraction solution, DPBS, Milli-Q water, and 1 vol% ethanol-water.

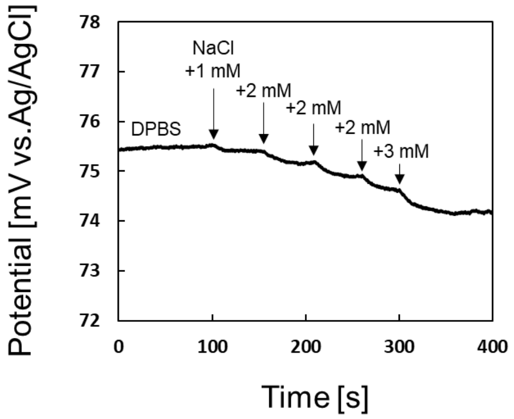

Figure S2.

Potentiometric response of a bare Ag/AgCl electrode against a commercially available Ag/AgCl reference electrode upon titration of NaCl into DPBS measurement solution. Totally, 10 mM of NaCl was added to the DPBS measurement solution.

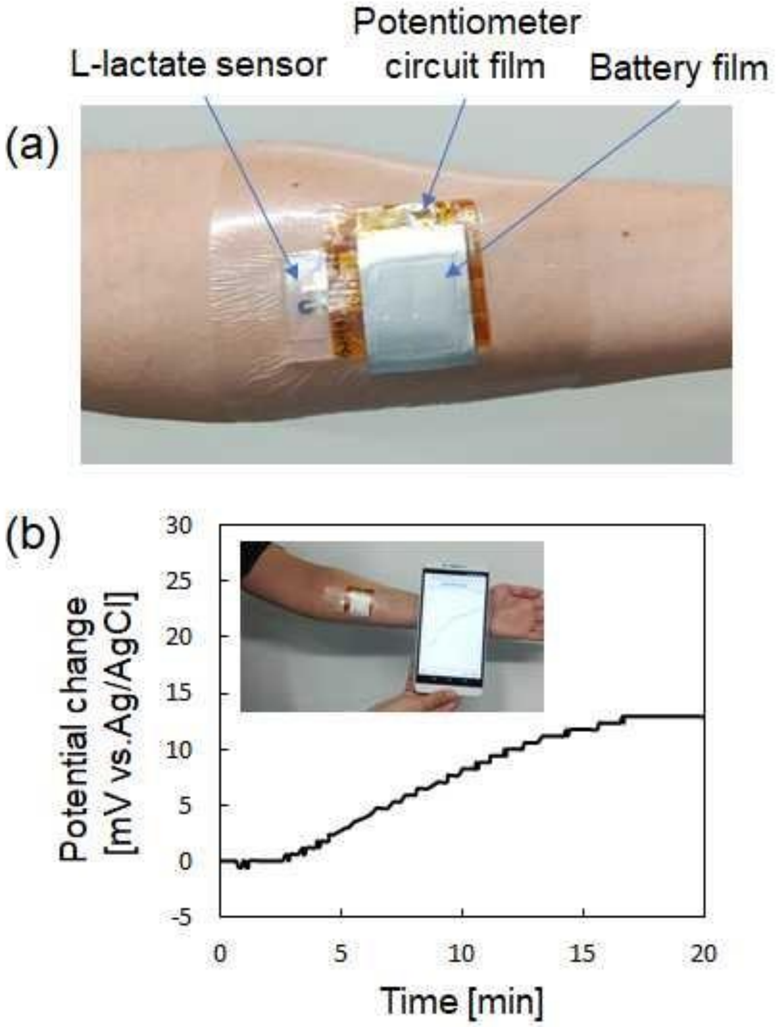

Figure S3.
(a) Photograph of the agarose gel-based L-lactate sensor combined with a potentiometer circuit and battery films. The sensing device was worn on a human forearm. (b) Realtime monitoring of the potentiometric response of L-lactate sensing device worn on a human forearm. Bluetooth Low Energy (BLE) protocols are used for the wireless communication as shown in the inserted photograph.

Supplementary Information

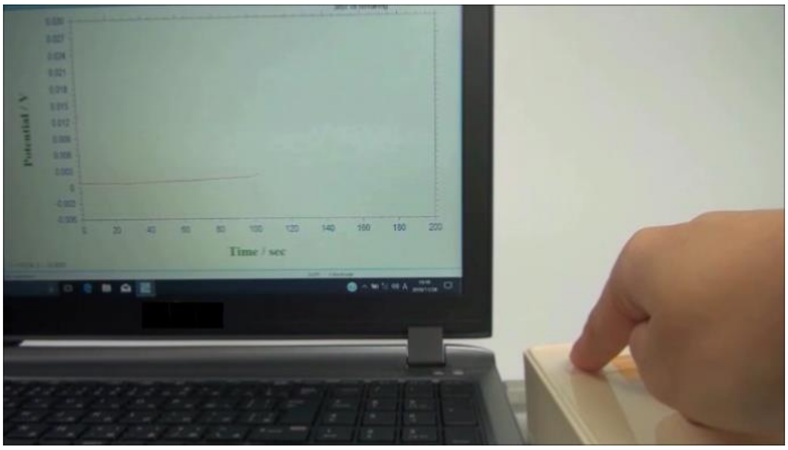

Movie S1

Movie of a human sweat extraction and detection experiment using the hydrogel-based L-lactate sensor. The movie was run in 4x speed.
